# Physical Exercise Repairs Obstructive Jaundice-Induced Damage to Intestinal Mucosal Barrier Function *via* H_2_S-Mediated Regulation of the HMGB1/Toll Like Receptors 4/Nuclear Factor Kappa B Pathway

**DOI:** 10.3389/fphys.2021.732780

**Published:** 2022-02-04

**Authors:** Changfeng Shao, Ye Li, Jiaqin Chen, Lan Zheng, Wei Chen, Qi Peng, Rui Chen, Afang Yuan

**Affiliations:** Hunan Provincial Key Laboratory of Physical Fitness and Exercise Rehabilitation, Hunan Normal University, Changsha, China

**Keywords:** aerobic exercise, hydrogen sulfide, obstructive jaundice, intestinal mucosal barrier, HMGB1/TLR4

## Abstract

The present study aimed to determine the effect of aerobic exercise on improving damage to intestinal mucosal barrier function caused by obstructive jaundice (OJ) and explore the mechanism. Fifty male KM mice were divided into five groups: sham operation group (S), model group (M), exercise group (TM), DL-propargylglycine + exercise (PT) group, and sodium hydrosulfide + exercise (NT) group. Additionally, mice in S group underwent common bile duct ligation for 48 h to establish a murine obstructive jaundice model. In PT group, propargylglycine (40 mg/kg) was intraperitoneally injected 7 days after surgery. NaHS (50 μmol/kg) was intraperitoneally injected into mice in the NT group 7 days after surgery. The TM group, NT group and PT group exercised on a slope of 0% at a speed of 10 m/min without weight training (30 min/day). HE staining showed that the intestinal mucosa of group M was atrophied and that the villi were broken. The intestinal mucosal structure of mice in the TM group was improved. Serum assays showed that H_2_S levels were higher in the TM group than in the M group; compared with the levels in the TM group, the PT group levels were decreased and the NT group levels were increased. In addition, aerobic exercise inhibits the HMGB1/TLR4/NF-κB signaling pathway by promoting endogenous H_2_S production, thereby exerting a protective effect on the intestinal mucosal barrier.

## Introduction

Obstructive jaundice (OJ) is a life-threatening disease that is common in hepatobiliary tissue (Ishizawa et al., 2007; Jin et al., 2019). Malignant tumors or stones can block the bile duct, leading to unsuccessful discharge of bile into the intestine (Dimas et al., 2019). In this situation, bile accumulates in the liver, and there is a reduction in bile in the intestine. The accumulation of bile induces biochemical, histological, and immunological changes that ultimately result in hepatic injury and even liver failure (Maeda et al., 2019). In addition, a lack of bile in the intestine injures the intestinal mucosal barrier, immunological barrier, and biochemical barrier (Banerjee et al., 2016). Once intestinal bacteria and endotoxins enter the blood circulation through the intestinal mucosa, bacterial translocation (BT), and endotoxin translocation (ET) occur (Zhou et al., 2018). BT is responsible for releasing inflammatory cytokines and stimulating immune cells, which contributes to systemic inflammation and even multiple organ dysfunction (Zhuang et al., 2019).

As endogenous ligands of Toll Like Receptors (TLR)-2/4, High Mobility Group Box 1 (HMGB1) regulates Nuclear Factor Kappa B (NF-κB) *via* multiple pathways and induces the release of inflammatory factors such as cytokines, chemokines and adhesion molecules, which results in the occurrence of inflammation (Liu et al., 2019). Additionally, HMGB1 plays a crucial role in the occurrence and development of intestinal ischemia-reperfusion injury (Wen et al., 2020). The HMGB1/TLR4/NF-κB pathway also participates in indomethacin- and acute pancreatitis-induced intestinal mucosal injury (Liu and Wang, 2019). However, changes in the HMGB1/TLR4/NF-κB pathway in the intestine in the context of OJ are still not well understood.

With chronic OJ, the liver and intestine are injured. Generally, even after surgery to correct the OJ, it is difficult to completely recover (Liu et al., 2021). Several studies have suggested that regular aerobic exercise is an easy method that has positive effects on chronic low-grade inflammation and restores cell proliferation and tissue repair (Batatinha et al., 2019a,b). In addition, aerobic exercise stimulated the expression of factors related to hepatocyte regeneration and proliferation, contributing to hepatocyte repair and regeneration (Ikeda et al., 2020). Moreover, aerobic exercise can exert curative effects on intestinal ischemia-reperfusion, inflammatory bowel disease and intestinal mucosal injury in colon cancer by decreasing several inflammatory factors, including HMGB1 and TLR4 (Miyake et al., 2020). H_2_S is an endogenous gaseous transmitter that, in addition to NO and CO, induces similar improvements in ameliorating the symptoms and factors described above (Cao et al., 2018). The main source of H_2_S is NaHS. NaHS is stable, and its concentration is easy to control (Niederauer et al., 2019). Thus, NaHS is widely used in the laboratory. Cystathionine-γ-lyase (CSE) is a synthetase for H_2_S that can be blocked by propargylglycine (PAG) to inhibit the synthesis of H_2_S (Matoba et al., 2020; Olson et al., 2020). The H_2_S/CSE signaling pathway can be upregulated by physical exercise (Wu et al., 2019). Thus, both physical exercise and H_2_S have positive effects on intestinal mucosal injury and depress the HMGB1/TLR4/NF-κB pathway. Physical exercise also increases H_2_S levels in organisms (Seifi et al., 2019). Unfortunately, the effects of aerobic exercise on OJ-induced intestinal mucosal injury, especially the molecular mechanism, are not well understood.

Based on previous evidence, we hypothesize that aerobic exercise inhibits the HMGB1/TLR4/NF-κB pathway by regulating H_2_S production in organisms. In the present study, the OJ model was established using mice, which were treated with CSE and NaHS. Aerobic exercise was also performed by the mice. Intestinal histology, liver function, serum factors (H_2_S, DAO, and D-lactate levels), and the expression of key genes (HMGB1, TLR4, and NF-κB p65) in the intestine were assayed. The present results suggest that aerobic exercise can regulate the concentration of endogenous H_2_S to affect the mucosal barrier in mice with OJ and describe the potential functional mechanisms.

## Materials and Methods

### Animals and Obstructive Jaundice Model

All animal experiments were approved by the Animal Care and Use Committee of Hunan Normal University (Changsha, China). Healthy adult male KM mice (body weight = 18–22 g) were provided by Hunan SJK Laboratory Animal Co., Ltd. (Changsha, China). The mice were housed in cages with 12 h light/12 h dark cycles at 23–25°C with 40–60% humidity and *ad libitum* access to food and sterile tap water. Fifty mice were divided into five groups: S, M, TM, PT, and NT groups (*n* = 10 in each group). Before model induction, the mice were housed for 1 week to adapt to the conditions. OJ was induced in mice in the M, TM, PT, and NT groups according to a previous study. Briefly, the mice were first anesthetized by intramuscular injections of ketamine (50 mg/kg) and xylazine (23 mg/kg). Subsequently, the common bile duct was ligated with a non-absorbable suture after midline laparotomy and exposure of the hepatic hilum. A part of the non-absorbable suture was retained outside of the body, which facilitated ease of removal. The individuals in the S group underwent midline laparotomy with no other operation. After 2 days, the non-absorbable suture was removed to reset the common bile duct.

### Treatment of Animals and Sampling

Five days after resetting the common bile duct, mice in the TM, PT, and NT groups were trained with treadmills (the slope was 0% and the speed was 10 m/min) for 30 min per day (Aoi et al., 2013). The training was performed 6 days per week and lasted for 6 weeks. Mice in the PT and NT groups were injected with PAG (40 mg/kg) and NaHS (50 μmol/kg) every day for 6 weeks. Mice in the M group were not injected.

After 6 weeks, the mice were fasted for 1 day and anesthetize with 1% pentobarbital sodium. Blood was collected from the posterior vena cava, and serum was isolated by centrifugation at 4,500 rpm for 15 min at 4°C. The serum was stored at −20°C until analysis. The terminal ileum was collected from the mice. The intestinal contents were washed with physiological saline and stored at −20°C. A part of the terminal ileum was collected and stored at −80°C for RNA and protein isolation. The other part of the terminal ileum was stored in 10% paraformaldehyde for histological analysis and immunohistochemical staining.

### Histological Analysis

The intestinal tissues were fixed in 10% paraformaldehyde and dehydrated with ethanol (from 70 to 100%). Subsequently, the tissues were treated with the hydrophobic clearing agent xylene and embedded in molten paraffin wax. The sections (thickness: 3 μm) were prepared and stained with hematoxylin and eosin for histological analysis under a bright-field Nikon Eclipse E800 microscope (Nikon, Japan).

### Determination of Serum Indicators

Total bilirubin (TBIL), alanine aminotransferase (ALT), and aspartate aminotransferase (AST) levels in the blood were determined by an automatic biochemical analyzer (AU5400TM; Olympus Optical Corp., Ltd., Japan) (*n* = 10 in each group). The concentrations of H_2_S in blood were measured using a spectrophotometer according to a previous study. Diamine oxidase (DAO) levels in blood were determined using commercial kits from QIYI Biological Technology Co., Ltd. (Shanghai, China), and D-lactic acid concentrations were determined by D-lactic acid assay kits from CNHAOBIO (Beijing, China) according to the manufacturer’s instructions.

### Immunohistochemical Staining

Immunohistochemical analysis was performed according to a previous study. Briefly, after dewaxing, rehydration and antigen retrieval by citrate-EDTA buffer, the sections were blocked with 5% goat serum for 10 min at 20°C. Then, the sections were incubated with HMGB1, TLR4 and NF-κB p65 antibodies (1:100) (Abcam, Cambridge, United Kingdom) for 1 h at room temperature. After being washed with PBS three times, the sections were stained with secondary antibodies (1:500) (Abcam, Cambridge, United Kingdom) for 30 min at room temperature, followed by three washes with PBS. The signals were detected by DAB solution (Biotinge Biomedicine Co., Ltd., Beijing, China). The sections were observed and recorded by a Nikon Eclipse E800 microscope (Nikon, Japan), and the area of the positive signals (*n* = 10 in each group) was calculated by C-Imaging software (SimplePCI, United States).

### Real-Time PCR

Total RNA was isolated from the intestines (*n* = 10 in each group) using TRIzol reagent (Invitrogen Life Technologies, Paisley, United Kingdom) according to the manufacturer’s instructions. The quality of the RNA was determined using 1.2% agarose gel electrophoresis. First strand cDNA was transcribed using the PrimeScript RT reagent kit with gDNA Eraser (TaKaRa, Dalian, China) from 1 μg total RNA. Using this kit, the genomic DNA was digested. The primers were designed by Primer Premier 5.0 based on the gene sequences from NCBI GenBank (Accession number: HMGB1, NM_001313894; TLR4, NM_021297; NF-κB, XM_006501106; GAPDH, NM_001289726). The primers are shown in Table 1. The PCR conditions included 95°C for 10 min for predenaturation, followed by 40 cycles of 95°C for 15 s, 60°C for 30 s, and 65°C for 30 s. The reactions contained 10 μl of 2 × TB Green Premix Taq II (Tli RNaseH Plus) (Takara, Dalian, China), 0.4 μl of each specific primer (10 μM), 2.0 μl of first-strand cDNA template and 7.2 μl of ddH_2_O and were processed on a CFX96 Real-Time PCR Detection System (BioRad). After the reaction, melt curve analysis was performed to confirm gene-specific amplification. In each group, 10 individuals were included in the experiment. Three biological replications were performed for each sample in the present study. The relative gene expression was calculated using the 2(-Delta Delta Ct) method (Rao et al., 2013). Glyceraldehyde 3-phosphate dehydrogenase (GAPDH) was used as a reference gene and internal control.

**TABLE 1:** The gene primers used in the present study.

Gene names	Primers	Product length (bp)
HMGB1	Forward 5′-GCTGACAAGGCTCGTTATGAA-3′	149
	Reverse 5′-CCTTTGATTTTGGGGCGGTA-3′	
TLR4	Forward 5′-TTCTGAGTAGCCGCTCTGGC-3′	139
	Reverse 5′-TGCCTCCCCAGAGCATTGTC-3′	
NF-κB	Forward 5′-CAGGTCCACTGTCTGCCTCT-3′	103
	Reverse 5′-GGAAGGATGTCTCCACACCA-3′	
GAPDH	Forward 5′-AATCTCCACTTTGCCACTGC-3′	191
	Reverse 5′-GTTTCCTCGTCCCGTAGACA-3′	

### Statistical Analysis

The data are shown as the average ± standard deviation and were analyzed using SPSS 19.0 (SPSS Inc., United States). Significant differences were determined using *t*-tests and were confirmed when *P* < 0.05.

## Results

### Survival Rate and Appearance

No mice died during the experiment. Twenty-four hours after surgery, the mice showed symptoms of narcolepsy, mental fatigue and bradykinesia. After surgery, the ha mice had messy and dull hair, with yellowish or reddish urine. Forty-eight hours after surgery, the tails and tip of the ears turned yellow. The feces from these mice were reduced and light in color. Several mice had symptom of constipation. Five days after resetting the common bile duct, the appearances of mice recovered, and the mice could perform low-intensity aerobic exercise. After 6 weeks of exercise, the appearances of the mice recovered. The effects were reduced in mice in the NT, TM, and PT groups.

### Histological Analysis of the Ileum

**FIGURE 1:**
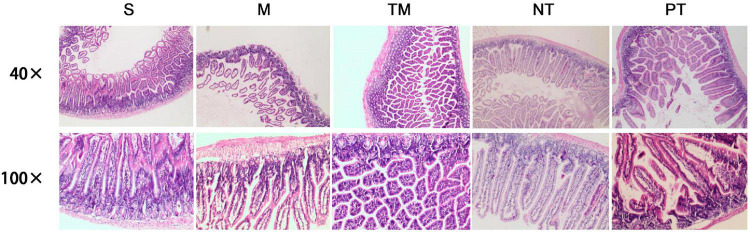
HE staining of pathological sections of mouse ileum in each group.

In the 40 × visual field, normal ileal tissues were observed in the S group. The intestinal villi of the M group were sparse with atrophy and gland abscission. Inflammatory cell infiltration was observed, especially basal lymphocyte infiltration. The muscular layers shrank, and several layers disappeared. In the TM group, the glands were irregularly arranged, and hyperplasia of glandular tissues was observed in the basal region. No edema and reduced inflammatory cell infiltration were observed in the TM group. The NT group showed recovery of the mucosa with normal gland distribution. In the basal region, hyperplasia of glandular tissues and a large amount of mucus were observed. The villi were irregularly arranged in the PT group, and hyperplasia of glandular tissues was observed in the basal region (Figure 1).

The results in the 100 × visual field were similar. Normal villi were observed, and the glandular epithelium was arranged in an orderly manner in the S group. Moreover, hyperplasia of glandular tissues in the basal region and complete basal membranes and muscular layers were observed in the S group. In contrast, vacuolar degeneration in glandular epithelial cells, shortened cells, lymphocytic infiltration, reduced cell numbers in the basal region and fibroid changes were observed in the M group. In the ileum in the TM group, the glands were arranged neatly, and the cells were normal in morphology with slight dark staining of the nuclei and several vacuoles. In the NT group, the villus morphology was similar to that of the normal ileum; however, oozing blood was observed in several parts. Irregular arrangement of the gland, mucous secretion from glandular epithelial cells and inflammatory cell invasion were observed in the PT group (Figure 1).

### Analysis of Serum Indices

The highest concentrations of TBIL, ALT, and AST were detected in the M group, while lower TBIL, ALT, and AST levels were observed in the M group (*P* < 0.05). Compared to those of the TM group, the TBIL, ALT, and AST concentrations were significantly decreased (*P* < 0.05). The TBIL, ALT, and AST levels were higher in the PT group than in the TM group (*P* < 0.05) (Table 2). The H_2_S concentrations in the S, M, and PT groups were lower than those in the TM group (*P* < 0.05). In addition, the H_2_S concentration was higher in the NT group than in the TM group (*P* < 0.01) (Table 2). The lowest DAO and D-lactate levels were found in the S group, while the M group had the highest levels of DAO and D-lactate. In addition, the TM group had lower levels of DAO and D-lactate than the M and PT groups (*P* < 0.01). The NT group had lower DAO and D-lactate concentrations than the TM group (*P* < 0.01) (Table 3).

**TABLE 2:** Comparison of serum total bilirubin (TBIL), alanine aminotransferase (ALT), and aspartate aminotransferase (AST) in each group.

Groups	AST (IU/L)	ALT (IU/L)	TBIL (IU/L)
S	50.89 ± 5.80	15.60 ± 4.49	8.05 ± 1.70
M	83.04 ± 6.14**	46.53 ± 10.90*	26.75 ± 3.96**
TM	35.79 ± 7.95^##^	21.71 ± 3.78^#^	17.40 ± 2.52^##^
NT	22.71 ± 0.75^&^	14.87 ± 1.95^&^	11.22 ± 1.44^&&^
PT	67.62 ± 5.28^&&^	26.82 ± 14.13^&^	25.71 ± 1.89^&&^

*P < 0.05, **P < 0.01: compared with S group; ^#^P < 0.05, ^##^P < 0.01: compared with M group; ^&^P < 0.05, ^&&^P < 0.01: compared with TM group.

**TABLE 3:** Comparison of serum H_2_S, diamine oxidase (DAO), and D-lactic acid contents in each group.

Groups	H_2_S (μmol/L)	DAO (U/L)	D-lactic acid (μmol/L)
S	39.50 ± 3.66	1.63 ± 0.19	7.54 ± 0.38
M	49.50 ± 2.87*	6.49 ± 0.37**	8.73 ± 0.17**
TM	54.65 ± 1.17^#^	3.91 ± 0.08^##^	8.00 ± 0.03^##^
NT	63.10 ± 2.87^&&^	2.92 ± 0.10^&&^	7.02 ± 0.23^&&^
PT	30.29 ± 0.74^&&^	4.96 ± 0.06^&&^	8.33 ± 0.08^&&^

*P < 0.05, **P < 0.01: compared with S group; ^#^P < 0.05, ^##^P < 0.01: compared with M group; ^&^P < 0.05, ^&&^P < 0.01: compared with TM group.

### Expression of High Mobility Group Box 1, Toll Like Receptors 4, and Nuclear Factor Kappa B p65 in Mice in the Different Groups

**FIGURE 2:**
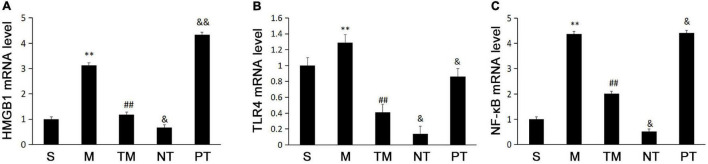
Expression of HMGB1 **(A)**, TLR4 **(B)**, and NF-κB p65, **(C)** mRNA in ileal tissue from each group of mice (*n* = 10). *P < 0.05, **P < 0.01: compared with the S group; #*P* < 0.05, ## < 0.01: compared with the M group; &*P* < 0.05, &&*P* < 0.01: compared with the TM group.

The real-time PCR results showed that the M group had higher mRNA expression of HMGB1 than the S group. Moreover, the TM group had lower HMGB1 mRNA expression than the M group (*P* < 0.01). The HMGB1 mRNA levels were higher in the TM group than in the NT group (*P* < 0.05) but were lower than those in the PT group (*P* < 0.01) (Figure 2A). TLR4 mRNA was highest in the M group, while the TM group had lower TLR4 mRNA levels than the M and PT groups (*P* < 0.01). In addition, the TLR4 mRNA level was lower in the NT group than in the TM group (*P* < 0.05) (Figure 2B). The lowest expression of NF-κB mRNA was found in the NT group, while the TM group had lower expression than the M group (*P* < 0.01). In the NT group, the NF-κB mRNA level was decreased compared to that of the TM group (*P* < 0.05) but increased in the PT group (*P* < 0.05) (Figure 2C).

**FIGURE 3:**
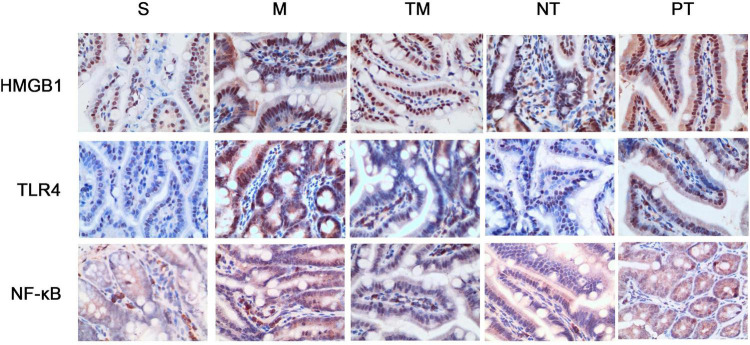
Immunohistochemical staining of HMGB1, TLR4, and NF-κB p65 in the ileum of each group of mice (400×).

**FIGURE 4:**
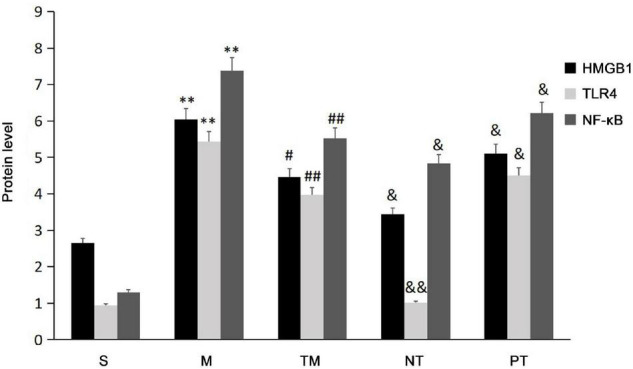
The positive signal areas in the different groups (*n* = 10). *P < 0.05, **P < 0.01: compared with the S group; #*P* < 0.05, ## < 0.01: compared with the M group; &*P* < 0.05, &&*P* < 0.01: compared with the TM group.

Protein expression was assayed using immunohistochemical staining. HMGB1 protein can be expressed in the nucleus and cytoplasm or secreted into the extracellular space. After physical exercise, the protein expression was decreased, and mice in the NT group had the lowest expression. TLR4 immunohistochemical staining showed that TLR4 was expressed in the cell membrane. In the S group, no significant TLR4 expression was detected, while the cells in the M group had obvious positive signals. After 6 weeks, TLR4 protein expression was decreased. The decrease in TLR4 protein expression was more significant in the TM and PT groups than in the NT group. The immunohistochemical staining results showed that NF-κB p65 was expressed in both the nucleus and cytoplasm. In cells from the S group, only slight staining was detected in the nucleus and cytoplasm, while obvious staining was observed in the nucleus in the M group. In cells from the TM and NT groups, only slight staining was found in the nucleus (Figure 3). The areas of the positive signals were calculated, and the S group had increased protein expression of HMGB1, TLR4, and NF-κB p65 (*P* < 0.01). The TM, NT, and PT groups had lower expression of these proteins than the M group. The NT group had lower HMGB1 and NF-κB p65 levels than the TM group (*P* < 0.05), and the lowest protein expression of TLR4 was found in the TM group (*P* < 0.01) (Figure 4).

## Discussion

Obstructive jaundice is mainly caused by partial or complete obstruction of extrahepatic or intrahepatic bile ducts, which results in the inability to discharge bile into the intestinal lumen (Kimmings et al., 2000). A lack of bile in the intestine inhibits bacterial reproduction and degrades intestinal endotoxins, resulting in intestinal mucosal stasis, edema, necrosis, villi atrophy, and mucosal thinning (Trauner and Boyer, 2003). Research on OJ-induced intestinal injury has received attention from clinical and basic researchers. Establishment of an ideal OJ animal model was key to the success of this experiment (Aoki et al., 2016). Model establishment is conducive to the study of not only drugs and other treatment options but also the cause of the disease. Previously, OJ models have been established mostly in rats (Ogata et al., 2003). In terms of surgical difficulty and survival rate, rats have certain advantages, but rats do not have a gallbladder. Thus, the physiological structure is quite different from that of humans. KM mice are more similar than rats to humans. In the present study, we used KM mice as a model to provide different information from the rat models. In addition, by using a relieved obstruction model, we simulated the effects of surgery in human OJ. One week after the obstruction was relieved, the survival rate was 94%, and low- to medium-intensity aerobic exercise could be performed. Chronic low-grade inflammation is an important risk factor that induces common chronic diseases (such as cardiovascular disease, diabetes, and cancer) (Henson et al., 2013). Regular exercise can prevent and treat these diseases (Radak et al., 2008). The possible mechanism by which exercise exerts anti-inflammatory effects by promoting angiogenesis in adipose tissue, improving the hypoxic state, promoting the phenotypic conversion of adipose tissue macrophages, inhibiting the release of inflammatory factors from adipose tissue, promoting the release of anti-inflammatory factors from skeletal muscle, and regulating the peripheral improvements in blood cell function (Gleeson et al., 2011). Aerobic exercise plays an important role in cell proliferation and tissue damage during inflammation (Petersen and Pedersen, 2005). A large number of studies have shown that long-term aerobic exercise can inhibit the apoptosis of cardiomyocytes, nerve cells, and skeletal muscle cells and promote cell proliferation (Song et al., 2006; Quadrilatero et al., 2011; Aoi et al., 2013). Previous studies have shown that regular aerobic exercise can improve OJ-induced liver injury, inhibit hepatocyte apoptosis induced by jaundice, and promote hepatocyte proliferation by regulating the expression of factors related to hepatocyte regeneration and delaying liver tissue fibrosis (Haczeyni et al., 2015).

Packer and Hoffman-Goetz (2012) found that long-term low-intensity aerobic exercise training could protect the intestinal mucosa from inflammatory bowel disease by reducing inflammatory cytokines, but there has been no report on the effects of aerobic exercise on OJ-induced intestinal injury. Morphological analysis of the intestinal mucosa in this study showed that the ileum of mice in the OJ group showed severe pathological damage, and intestinal mucosal injury was significantly reduced in mice that underwent exercise training, indicating that 6 weeks of aerobic exercise exerted a significant protective effect on the intestinal mucosal barrier after OJ.

Diamine oxidase is an intracellular enzyme that is mainly distributed in the apical cells of the intestinal villi (Wang et al., 2016). DAO has the highest activity in the small intestine. When the intestinal mucosa is injured, the epithelium is destroyed, causing DAO to be released into the blood, and DAO activity in the blood increases (Zhang et al., 2002); thus, these changes can reflect the damage and repair of the intestinal mucosal barrier (Zhang et al., 2019). When intestinal injury causes the apical epithelium of the intestinal villi to fall off, the permeability of the intestinal mucosa increases, a large amount of D-lactic acid in the intestine enters the blood through the damaged intestinal mucosa, and the blood D-lactic acid level increases (Modi et al., 2006); thus, blood D-lactic acid levels can reflect the degree of intestinal mucosal injury and permeability changes. To further examine the degree of intestinal mucosal barrier damage, this study measured serum DAO and D-lactic acid levels. The results showed that serum DAO and D-lactic acid levels of mice in each group were ranked as follows: M group > PT group > TM group > NT group > S group; these results indicate that the mouse intestinal mucosal barrier was damaged after OJ, and aerobic exercise had obvious protective effects on the intestinal mucosal barrier.

It has been reported that hepatic reticuloendothelial cell dysfunction and damage to intestinal mucosal barrier function caused by OJ can lead to the production of proinflammatory cytokines, which are key to systemic inflammation and the cause of multiple organ dysfunction in OJ patients (Tian et al., 2019). Therefore, alleviating intestinal mucosal barrier damage and reducing the inflammatory response and the release of inflammatory cytokines have become key to the treatment of OJ-induced intestinal injury (Turner, 2009). HMGB1 is an important late inflammatory mediator. On the one hand, HMGB1 can be secreted by various nucleated cells, such as intestinal epithelial cells and immune cells activated by macrophages and monocytes (Tang et al., 2007). On the other hand, HMGB1 can also be passively released from damaged or necrotic cells to the peripheral blood circulation or outside the cell (Bianchi and Manfredi, 2007). Many recent studies of HMGB1 receptor and signal transduction pathways have confirmed that HMGB1 is an important endogenous ligand of TLR-2/4 (Liu and Wang, 2019; Wen et al., 2020). After HMGB1 and TLR-2/4 interact, NF-κB is activated through the MyD88-dependent pathway and the MyD88-independent pathway (Liu and Wang, 2019). After binding, NF-κB is activated through the MyD88-dependent pathway and the MyD88-independent pathway (Wang et al., 2015). The activation of NF-κB is downstream of TLR4 and can upregulate the expression of inflammatory factors and chemokines, such as TNF-α, IL-6, iNOS, and ICAM (Asehnoune et al., 2005). In addition, NF-κB is also related to cell apoptosis, survival, growth, and division (Lin and Karin, 2003). Animal models and a number of clinical studies have shown that HMGB1, TLR4, and NF-κB are involved in the process of intestinal mucosal injury caused by intestinal ischemia-reperfusion injury, indomethacin and acute pancreatitis, all of which play important roles (Nadatani et al., 2012; Chen et al., 2017; Lee and Papachristou, 2019; Han et al., 2020).

In this study, the relative mRNA expression levels of HMGB1, TLR4, and NF-κB in the M group were higher than those in the S group, indicating that these genes were significantly upregulated after biliary obstruction. Immunohistochemical staining showed that after biliary obstruction, the expression levels of HMGB1, TLR4, and NF-κB in the intestinal mucosa also increased. This finding further showed that the HMGB1/TLR4/NF-κB pathway was involved in OJ-induced intestinal mucosal epithelial cell injury. The findings of this study suggest that complete biliary obstruction, as a predisposing factor of intestinal mucosal injury, is similar to the mechanism of action of other damaging factors associated with intestinal injury and occur with the participation of the HMGB1/TLR4/NF-κB signaling pathway (Wang et al., 2015; Liu and Wang, 2019). Namely, the activation of the HMGB1/TLR4/NF-κB signaling pathway leads to damage to intestinal epithelial cells and the intestinal mucosal barrier.

As a newly discovered endogenous gaseous signaling molecule, H_2_S can be generated by the bacterial flora in the intestinal lumen and is closely related to a variety of intestinal diseases. However, research on the relationship between H_2_S and intestinal diseases is still in its infancy. The main sources of H_2_S are sodium hydrosulfide, sodium thiosulfate and sodium sulfide. This study used NaHS to generate H_2_S. NaHS can be dissociated into HS^–^ and Na^+^ in the body. HS^–^ interacts with H^+^ in the body to generate H_2_S. A dynamic equilibrium is formed between the two forms, which ensures the stability and accuracy of the H_2_S concentration in solution so that H_2_S exists stably without changing the pH value of the internal environment. The H_2_S synthase CSE can be blocked by PAG, and this blocking effect is irreversible, thereby inhibiting the production of H_2_S (Cao et al., 2018; Wu et al., 2019). Study has shown that H_2_S can improve intestinal ischemia-reperfusion, inflammatory bowel disease and intestinal mucosal injury (Nicholson and Calvert, 2010). Exercise training could restore plasma H_2_S levels and increase aortic CSE mRNA levels (Yang et al., 2006). Therefore, exercise training increases the production of endogenous H_2_S. In addition, aerobic exercise and H_2_S reduce the expression of HMGB1, TLR4, and other inflammatory factors. However, whether sports exert this effect through H_2_S has not yet been reported.

The results of this study showed that the level of H_2_S in the TM group was significantly increased, while the relative mRNA and protein expression levels of HMGB1, TLR4, and NF-κB were significantly lower than those in the M group, indicating that aerobic exercise can increase H_2_S levels. The expression of HMGB1, TLR4, and NF-κB in intestinal mucosal epithelial cells was reduced. Expression changes of intestinal genes may lead to multiple effects on organisms (Zhang et al., 2018; Zhou et al., 2019). The expression of the abovementioned genes and proteins in the NT group was significantly higher than that in the TM group, and the expression in the PT group was significantly lower than that in the TM group. These results suggest that after H_2_S is blocked by PAG, the expression of HMGB1-TLR4-NF-κB pathway-related factors is upregulated and reduces the protection of the intestinal mucosal barrier. Conversely, the use of the H_2_S donor NaHS inhibited the expression of HMGB1-TLR4-NF-κB pathway-related factors and improved intestinal mucosal barrier function. Therefore, it is hypothesized that aerobic exercise increases the level of endogenous H_2_S, thereby inhibiting the expression of HMGB1-TLR4-NF-κB signaling pathway-related factors and reducing the damage to the intestinal mucosa in mice with OJ, thereby protecting intestinal mucosal barrier function.

## Conclusion

In conclusion, the intestinal mucosal epithelium of mice with OJ is seriously damaged due to the inability of bile to pass into the intestinal lumen. Six weeks of low-to-medium-intensity treadmill exercise alleviated intestinal mucosal epithelial injury in mice with OJ and had an apparent protective effect on the intestinal mucosa barrier. The mechanism may be related to aerobic exercise increasing the level of endogenous H_2_S in OJ mice and inhibiting the expression of related factors in the HMGB1-TLR4-NF-κB signaling pathway.

## Data Availability Statement

The original contributions presented in the study are included in the article/supplementary material, further inquiries can be directed to the corresponding authors.

## Ethics Statement

The animal study was reviewed and approved by the Animal Care and Use Committee of Hunan Normal University. Written informed consent was obtained from the owners for the participation of their animals in this study.

## Author Contributions

CS, YL, and JC performed the investigation, analyzed the data, and wrote the original draft. YL, WC, and QP performed the experimental design and methodology. LZ, RC, and AY revised and edited the manuscript. All authors read and approved the final manuscript.

## Conflict of Interest

The authors declare that the research was conducted in the absence of any commercial or financial relationships that could be construed as a potential conflict of interest.

## Publisher’s Note

All claims expressed in this article are solely those of the authors and do not necessarily represent those of their affiliated organizations, or those of the publisher, the editors and the reviewers. Any product that may be evaluated in this article, or claim that may be made by its manufacturer, is not guaranteed or endorsed by the publisher.
